# Assessment of a deep learning model for COVID-19 classification on chest radiographs: a comparison across image acquisition techniques and clinical factors

**DOI:** 10.1117/1.JMI.10.6.064504

**Published:** 2023-12-28

**Authors:** Mena Shenouda, Isabella Flerlage, Aditi Kaveti, Maryellen L. Giger, Samuel G. Armato

**Affiliations:** a The University of Chicago, Committee on Medical Physics, Department of Radiology, Chicago, Illinois, United States; b Vanderbilt University, Nashville, Tennessee, United States; c Stony Brook University, Stony Brook, New York, United States

**Keywords:** COVID-19, deep learning, generalizability, chest radiography

## Abstract

**Purpose:**

The purpose is to assess the performance of a pre-trained deep learning model in the task of classifying between coronavirus disease (COVID)-positive and COVID-negative patients from chest radiographs (CXRs) while considering various image acquisition parameters, clinical factors, and patient demographics.

**Methods:**

Standard and soft-tissue CXRs of 9860 patients comprised the “original dataset,” consisting of training and test sets and were used to train a DenseNet-121 architecture model to classify COVID-19 using three classification algorithms: standard, soft tissue, and a combination of both types of images via feature fusion. A larger more-current test set of 5893 patients (the “current test set”) was used to assess the performance of the pretrained model. The current test set contained a larger span of dates, incorporated different variants of the virus and included different immunization statuses. Model performance between the original and current test sets was evaluated using area under the receiver operating characteristic curve (ROC AUC) [95% CI].

**Results:**

The model achieved AUC values of 0.67 [0.65, 0.70] for cropped standard images, 0.65 [0.63, 0.67] for cropped soft-tissue images, and 0.67 [0.65, 0.69] for both types of cropped images. These were all significantly lower than the performance of the model on the original test set. Investigations regarding matching the acquisition dates between the test sets (i.e., controlling for virus variants), immunization status, disease severity, and age and sex distributions did not fully explain the discrepancy in performance.

**Conclusions:**

Several relevant factors were considered to determine whether differences existed in the test sets, including time period of image acquisition, vaccination status, and disease severity. The lower performance on the current test set may have occurred due to model overfitting and a lack of generalizability.

## 1 Introduction

Severe acute respiratory syndrome coronavirus 2 (SARS-CoV-2), a ribonucleic acid (RNA) virus that can impact mammals and birds, is the virus responsible for the ongoing coronavirus disease 2019 (COVID-19) global pandemic. The primary mode of transmission among humans is through exposure to respiratory fluids carrying infectious virus. The virus is highly contagious and can rapidly mutate. Further, infection with the virus may lead to severe or fatal disease. Early detection of the disease can mitigate the symptoms, however, and patient prognosis can improve. Chest radiographs (CXRs) were recommended early in the pandemic for triage, disease monitoring, and assessment of concomitant lung abnormalities (e.g., consolidation, ground-glass opacities, and pulmonary nodules),^1^^,^^2^ which resulted in the acquisition of many medical images worldwide. CXRs were also beneficial as they are widely accessible, which make them an ideal modality for an image-based evaluation of the disease.

With the onset of the COVID-19 pandemic, the artificial intelligence (AI) community quickly joined in the effort to ease the burden on healthcare systems. Before widespread access to reverse transcription polymerase chain reaction (RT-PCR) tests, machine and deep learning (DL) models were developed to provide rapid COVID-19 diagnoses and prognoses based on patients’ CXRs and computed tomography scans.^3^^–^^8^ While many DL models reported success in performing these tasks, translating this success to a clinical environment has been difficult due to potential model overfitting or biases present in the datasets.^9^ These biases may result in a lack of reproducibility and generalizability of the models developed, a common shortcoming recognized by the AI community.^10^ The diversity of images acquired throughout the pandemic, therefore, allows for a comprehensive evaluation of AI models to assess the models’ generalizability when using the various datasets available.

To determine the robustness of a DL model, an independent dataset can be used along with a performance assessment metric [e.g., area under the receiver operating characteristic curve (ROC AUC)].^11^ If the performance on independent test sets is comparable to the performance on the original test set, then the model is deemed robust. If the model’s performance were to decrease, however, then further evaluation is warranted to determine possible deficiencies. While CXRs are not considered a clinical standard for COVID-19 diagnosis, their value lies in their utility for AI assessment. Therefore, the implementation of the DL models in this work is not intended for eventual clinical deployment but rather as a means to thoroughly evaluate the fundamentals of AI as a diagnostic tool.

Overall, the purpose of this work was to validate a DL model, using COVID-19 diagnosis from CXRs as the radiologic task, and to compare performance on different datasets while taking into consideration factors, such as image-acquisition device [e.g., portable units versus stationary dual-energy subtraction (DES) units], patient vaccination status, patient age, and disease severity.^12^ These analyses were meant to assess and quantify AI generalizability in the differentiation of COVID-positive and COVID-negative patients based on chest radiography. This research highlights the importance of not only developing DL models but also rigorously testing their performance across various scenarios to ensure robustness.

## 2 Methods

### 2.1 Datasets

#### 2.1.1 Original dataset

The original dataset consisted of 9860 patients retrospectively collected from the University of Chicago Medicine. This cohort was split at the patient level into 64% for training, 16% for validation, and 20% for testing using stratified sampling. COVID-19 prevalence was held constant across the three subsets (15.5%). Only the first CXR exam acquired within 2 days after a patient’s initial RT-PCR test for the SARS-CoV-2 virus was input to the model. CXRs had been acquired between January 30, 2020 and February 3, 2021; both standard and soft-tissue images were collected from stationary DES radiography units and portable radiography units (i.e., CXR exam type). The portable units generated soft-tissue images using post-processing algorithms. More information regarding this dataset can be found in Hu et al.;^13^ the test set from this study will be called the “original test set.”

#### 2.1.2 Current test set

Images that comprised the “current test set” were retrospectively collected from 5893 patients between March 15, 2020 and January 1, 2022 under a Health Insurance Portability and Accountability Act (HIPAA)-compliant, Institutional Review Board-approved protocol. Among these patients, 731 (12.4%) had tested positive and 5162 (87.6%) had tested negative for the SARS-CoV-2 virus. The current test set served only to assess the performance of the pre-trained model: no additional training or validation was performed. Patient images from the previous study and the current study were acquired from the same institution and were preprocessed in the same manner. Overall, the current test set followed the same curation process as the original dataset to minimize the impact of any confounding variables. A summary of the datasets is presented in Tables 1 and 2, where Table 2 categorizes the three manufacturers that were used to acquire the standard CXR images for both test sets: Canon Inc., GE Healthcare, and Fujifilm Corporation.

**Table 1:** Summary of datasets used, categorized by various factors, including type of units used to acquire the images.

	Number of patients	Average date of acquisition	COVID prevalence	Number of portable scans (%)	Number of DES scans (%)
Original training set	7888	August 12, 2020	15.4%	6243 (79.1%)	1645 (20.9%)
Original test set	1972	August 13, 2020	15.5%	1595 (80.1%)	377 (19.1%)
Current test set	5893	March 19, 2021	12.4%	4165 (71%)	1728 (29%)

**Table 2:** Number of patients categorized by manufacturer and CXR exam type for the original and current test set.

	Original test set		Current test	
	Portable	DES	Portable	DES
Canon Inc.	1595	12	4163	43
GE Healthcare	0	359	2	1596
Fujifilm Corporation	0	6	0	89
Total	1595	377	4165	1728

### 2.2 Image Preprocessing

The original digital imaging and communications in medicine (DICOM) images were gray-scale normalized per image (i.e., per sample) and converted to Portable Network Graphics (PNG) format. Using the converted PNG images, an open-source U-Net-based model was used to segment the lung region on radiographs from the original dataset and the current test set.^14^ The smallest rectangular region that contained the resulting lung mask on a patient’s standard CXR image then was cropped; the same mask was applied to a patient’s corresponding soft-tissue image. The weights used for the segmentation task were calculated using a pre-pandemic public CXR dataset^15^ and further fine-tuned on another dataset of radiographs displaying COVID-19.^13^^,^^16^ Cropping was shown to improve results on the original dataset^13^ and was, therefore, performed on the current test set to be (1) consistent and (2) ensure the DL model would not consider areas outside the outside the lungs (e.g., abdominal region, chest wall, shoulder, and neck region).

**Fig. 1:**
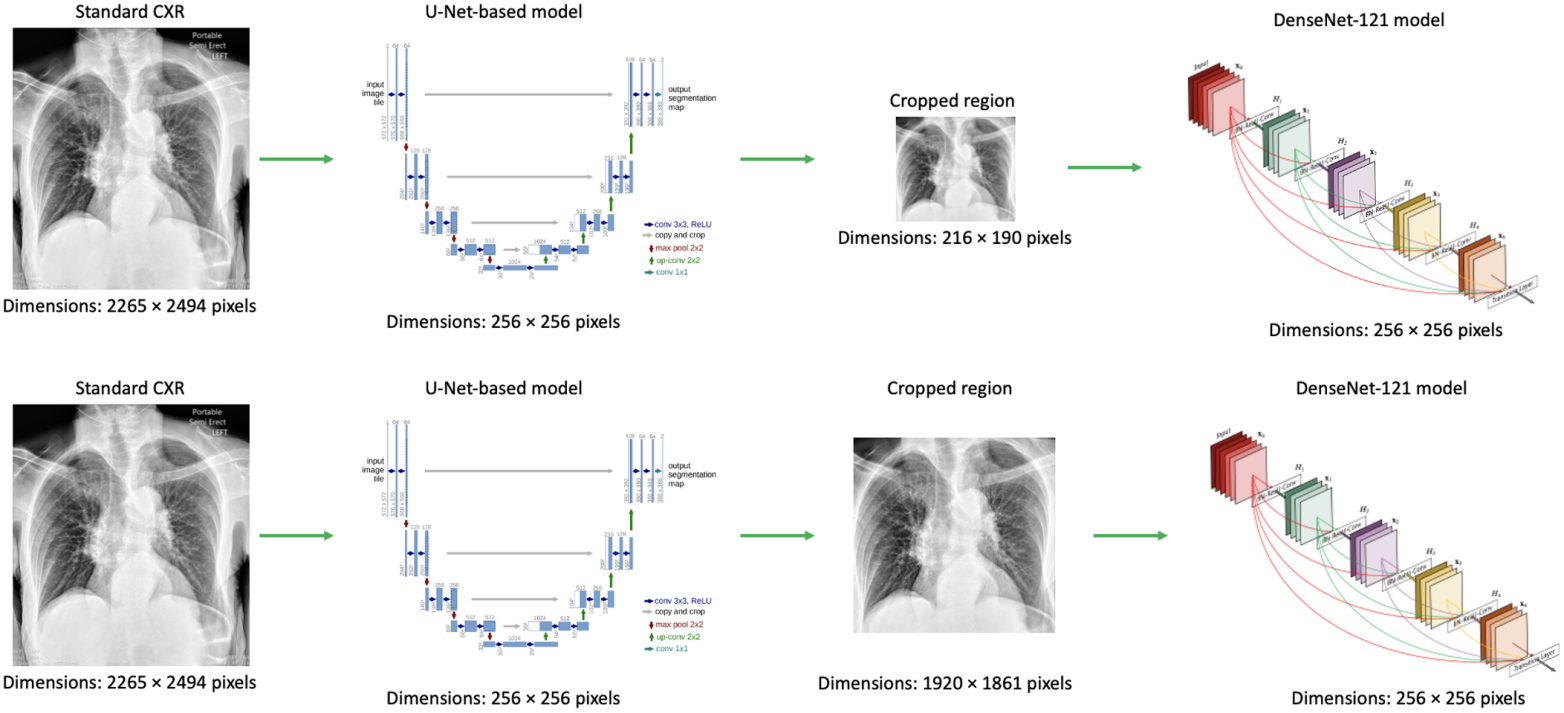
The image preprocessing pipeline used throughout this work. Top panel: a patient’s standard CXR was resized to 256×256  pixels, the lung region was subsequently segmented and cropped, which generated a rectangular region containing only the lung (the small lung region). Bottom panel: after the segmentation task, the cropping was performed in the same dimensional space as the original image.

The impact of the cropped lung region dimensions on the performance of the classification task was explored on the standard CXRs of the original test set. The U-Net-based model used for lung segmentation initially resized an entire image to 256×256  pixels and cropped the rectangular region that enclosed the predicted lung mask, which will be called the “small lung region.” This small lung region was then upsampled to 256×256  pixels by the DenseNet-121 model^17^ prior to classification (top panel of Fig. 1). To study the impact of image resizing, two investigations were performed. First, the U-Net-based model was adjusted to generate the segmented lung region in the same dimensional space as the original image. The resultant “large lung region” image was then input to the DenseNet-121 model for classification (bottom panel of Fig. 1). The second investigation resized the large lung region image in the bottom panel of Fig. 1 to the size of the corresponding small lung region image in the top panel of Fig. 1 before the classification step. All image resizing was performed using the Python PIL package “Image” module, which is also utilized by the U-Net-based model.

### 2.3 Model Implemented

The basis for the present study was a model that was developed by Hu et al.^13^ using the original dataset. The model was based on a DenseNet-121 architecture because of its previous success in diagnosing pneumonia and other pathologies on CXRs.^18^^,^^19^ Further, it followed a curriculum learning methodology as discussed in Bengio et al.^20^ The curriculum learning became more specialized at each phase, concentrating the model on COVID-19 by the last step. The three phases were: (1) pre-train on ImageNet and fine-tune on the National Institutes of Health (NIH) ChestX-ray14,^21^^,^^22^ (2) refine on the Radiological Society of North America Pneumonia Detection Challenge dataset to detect pneumonia,^23^ and (3) further train on an in-house COVID-19 dataset. Specifically, phase 3 consisted of three classification algorithms developed by image type: standard, soft-tissue, and a combination of both image types via feature fusion, as presented by Hu et al.^13^ Throughout this study, the algorithms were applied to their corresponding image type (e.g., the algorithm developed with standard CXRs was tested only on standard CXRs). All of the statistical analyses focused on the performance of the current test set when input to the pre-trained model after phase 3, with no additional training or validation performed.

### 2.4 Statistical Analysis

#### 2.4.1 Current test set only

Performance of the three classification algorithms was assessed by CXR exam type (portable versus DES) on the current test set.

#### 2.4.2 Original test set versus current test set

Comparisons of model performance between the original and current test sets were performed for standard CXRs when considering (1) the entirety of the two test sets (this comparison was repeated for the soft-tissue CXRs and fusion of the image types), (2) the original test set and only CXRs from the current test set acquired within the date range of the original test set (to control for the different virus variants), and (3) nonimmunized patients from both test sets (to control for the impact of disease severity due to vaccines).

The ability of the DL model to classify disease will depend on the severity of the disease as presented on a medical image. Therefore, the COVID severity of the CXRs in the original and current test sets was calculated, as studied by Li, et al.^24^ Briefly, the COVID severity model computed a pulmonary x-ray severity (PXS) score, which is defined as the median Euclidean distance between the image of interest and “normal” images (i.e., absence of all pathologies^25^). Specifically, the Euclidean distance, with respect to the imaging features on which the networks trained, was between the final two layers of twinned DenseNet-121 networks within a Siamese neural network. On a subset of 50 cases from the original test set chosen using stratified sampling, the manual modified radiographic assessment of lung edema scores (mRALE, based on the RALE score created by Warren et al.^26^) were determined by a radiologist with over 20 years of experience. The PXS scores were assessed using a Bland–Altman plot^27^ to display the agreement between the computed PXS and mRALE scores. The Spearman’s rank correlation coefficient was also calculated to assess the monotonic relationship between the PXS and mRALE scores. Model performance based on PXS score was evaluated after grouping the cases into four equally spaced bins. Due to the small numbers in the fourth bin for each of the test sets, however, the cases for the third and fourth bins were combined, resulting in three bins for analysis. The “obviousness” of each case was also qualitatively assessed by plotting the DL prediction scores of the cropped standard images from the original and current test sets.

Uniform manifold approximation projection (UMAP^28^) was used to visualize the penultimate global average pooling layer of the standard model to qualitatively evaluate the COVID-19 classification task and to visualize the confusion matrix. A quantitative comparison of the UMAPs was performed using one-way multivariate analysis of variance (MANOVA) to test for a significant difference between the two bivariate means of the UMAPs; specifically, the bivariate means were tested for statistical significance using Pillai’s trace to calculate the F-statistic, which then resulted in its corresponding p-value. In addition, a comparison of demographics (i.e., age and sex distributions) between patient cohorts was performed, and an analysis of standard model performance with images of the various image dimensions previously described of the image preprocessing steps was conducted on the original test set.

Overall, these investigations were designed to better understand potential confounding factors that might have occurred during image acquisition, different variants of the COVID-19 virus, and patient age. Furthermore, clinical factors, such as vaccination status and severity of COVID, were assessed. Performance was evaluated using area under the ROC curve as the figure of merit (2000 bootstrapped samples to construct the 95% confidence intervals), with the DeLong test used to compare the uncorrelated ROC curves.^29^

## 3 Results

### 3.1 Current Test Set Only

#### 3.1.1 Classification algorithm

**Fig. 2:**
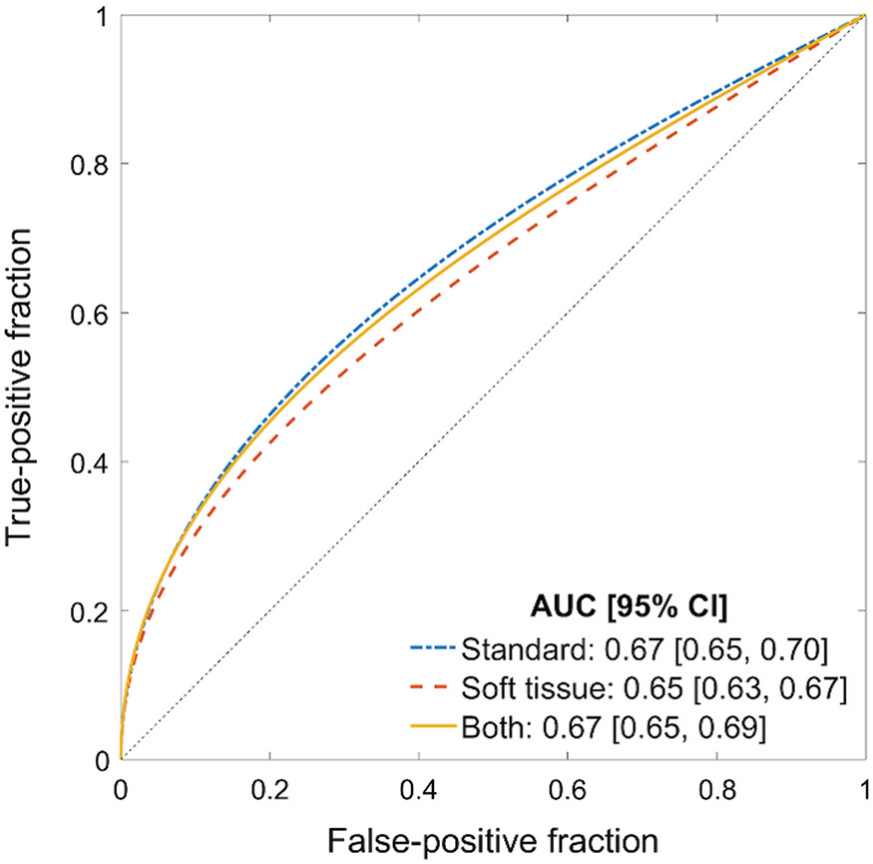
ROC curves for the classification of COVID-positive and COVID-negative patient CXRs using the current test set. No additional training or validation was performed. ROC curves were fitted using software as defined by Metz et al.^31^

The three classification algorithms corresponded to training the model with the original dataset using (1) cropped standard images, (2) cropped soft-tissue images, (3) and a feature fusion of the two image types. The pre-trained (i.e., no additional training or validation) DL model then was applied to the current test set and achieved the AUC values presented in Fig. 2.

The AUC value obtained using the cropped standard CXR images (0.67 [0.65, 0.70]) was significantly higher than that obtained using soft-tissue CXRs (0.65 [0.63, 0.67]). AUC values, however, failed to achieve statistical significance when comparing (1) cropped standard CXRs with both types of images (fusion) and (2) soft-tissue CXRs with both types of images. The results are displayed in Table 3.

**Table 3:** Comparisons of model performance for the three classification algorithms: standard images, soft-tissue images, or both types. P-values comparing the differences in AUC values were calculated using the DeLong test, with their corresponding confidence intervals (CIs), prior to multiple comparisons correction.^30^ Significance levels (α) and widths of the CIs were adjusted based on multiple comparisons.

	P-value for ΔAUC	α	CI of AUC
Standard versus soft-tissue	0.0069^a^	0.017	98.3% CI = [0.0030, 0.050]
Standard versus fusion	0.42	0.050	95% CI = [−0.010, 0.025]
Soft-tissue versus fusion	0.031	0.025	97.5% CI = [−0.039, 0.00075]

a Statistically significant difference after correcting for multiple comparisons (Bonferroni–Holm correction).

#### 3.1.2 CXR exam type

When considering the CXR exam type (e.g., portable versus a DES unit), the AUC values achieved are shown in Table 4. AUC values failed to achieve statistical significance when comparing across CXR exam type for each classification algorithm, i.e., the type of unit did not appear to have an impact on the model’s performance in the task of classifying COVID-19.

**Table 4:** Model performance categorized by the different image types (classification algorithms) as acquired from the different radiography units for the current test set. 95% CIs are displayed in brackets. Majority of portable images were acquired on Canon Inc. units and majority of DES images were acquired on GE Healthcare and Fujifilm Corporation units.

		Portable = 4165 (70.7%)	DES = 1728 (29.3%)	Overall = 5893
COVID-19 prevalence		471 (11.3%)	260 (15.0%)	731 (12.4%)
AUC [95% CI]	Standard	0.68 [0.65, 0.71]	0.69 [0.65, 0.73]	0.67 [0.65, 0.70]
	Soft-tissue	0.66 [0.63, 0.69]	0.64 [0.60, 0.68]	0.65 [0.63, 0.67]
	Fusion	0.68 [0.65, 0.71]	0.68 [0.64, 0.72]	0.67 [0.65, 0.69]

### 3.2 Original Test Set Versus Current Test Set

#### 3.2.1 Entirety of both test sets

**Fig. 3:**
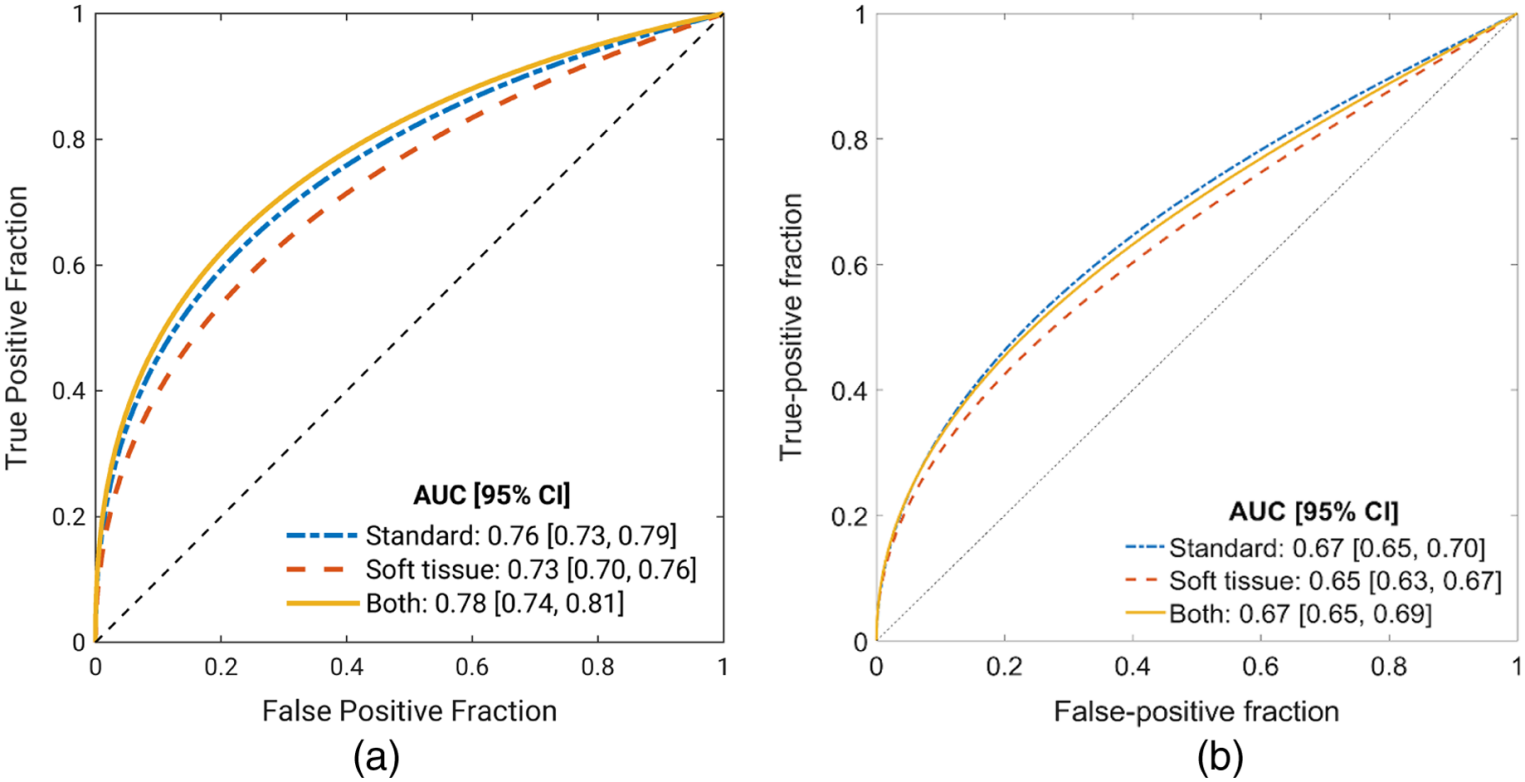
Comparison of ROC curves and AUC values between (a) original and (b) current test sets. AUC values for the three classification algorithms were consistently lower for the current test set compared with the original. Panel (a) is the same figure as Fig. 6 in Ref. 13 (reprinted with permission) and panel (b) is the same as Fig. 2.

All AUC values obtained in Fig. 2 were lower than those obtained in Hu, et al.^13^ (p<0.001) as calculated using the DeLong test for uncorrelated ROC curves. Figure 3 shows the comparison of ROC curves and values between the two test sets.

#### 3.2.2 Date match

There were four main COVID-19 variants of concern (VOC) that underlie the datasets (as defined by the City of Chicago Department of Public Health):^32^ the original strain, B.1.1.7 (Alpha) beginning in January 2021 until June 2021, Delta from July 2021 to December 2021, and Omicron in December 2021 onward. Therefore, the original dataset had two VOC: the original variant and Alpha. The current test set had all four VOC. Table 5 displays the AUC values acquired when controlling for the variants.

**Table 5:** AUC values calculated for each of the four variants underlying the two test sets.

	Original variant (start to December 31, 2020)	Alpha (January 1, 2021 to June 30, 2021)	Delta (July 1, 2021 to November 30, 2021)	Omicron (December 1, 2021 to present)
Original test set AUC [95% CI]	0.77 [0.73, 0.80]	0.65 [0.52, 0.79]	NA	NA
Current Test Set AUC [95% CI]	0.67 [0.62, 0.71]	0.68 [0.65, 0.72]	0.71 [0.66, 0.76]	0.63 [0.54, 0.71]
Number of patients in original test set (COVID prevalence)	N=1782 (15.9%)	N=190 (11.1%)	N=0	N=0
Number of patients in current test set (COVID prevalence)	N=1552 (14.4%)	N=2827 (10%)	N=1320 (11.6%)	N=194 (36.6%)
AUC for variant	0.72 [0.70, 0.75]	0.68 [0.65, 0.72]	NA	NA

**Fig. 4:**
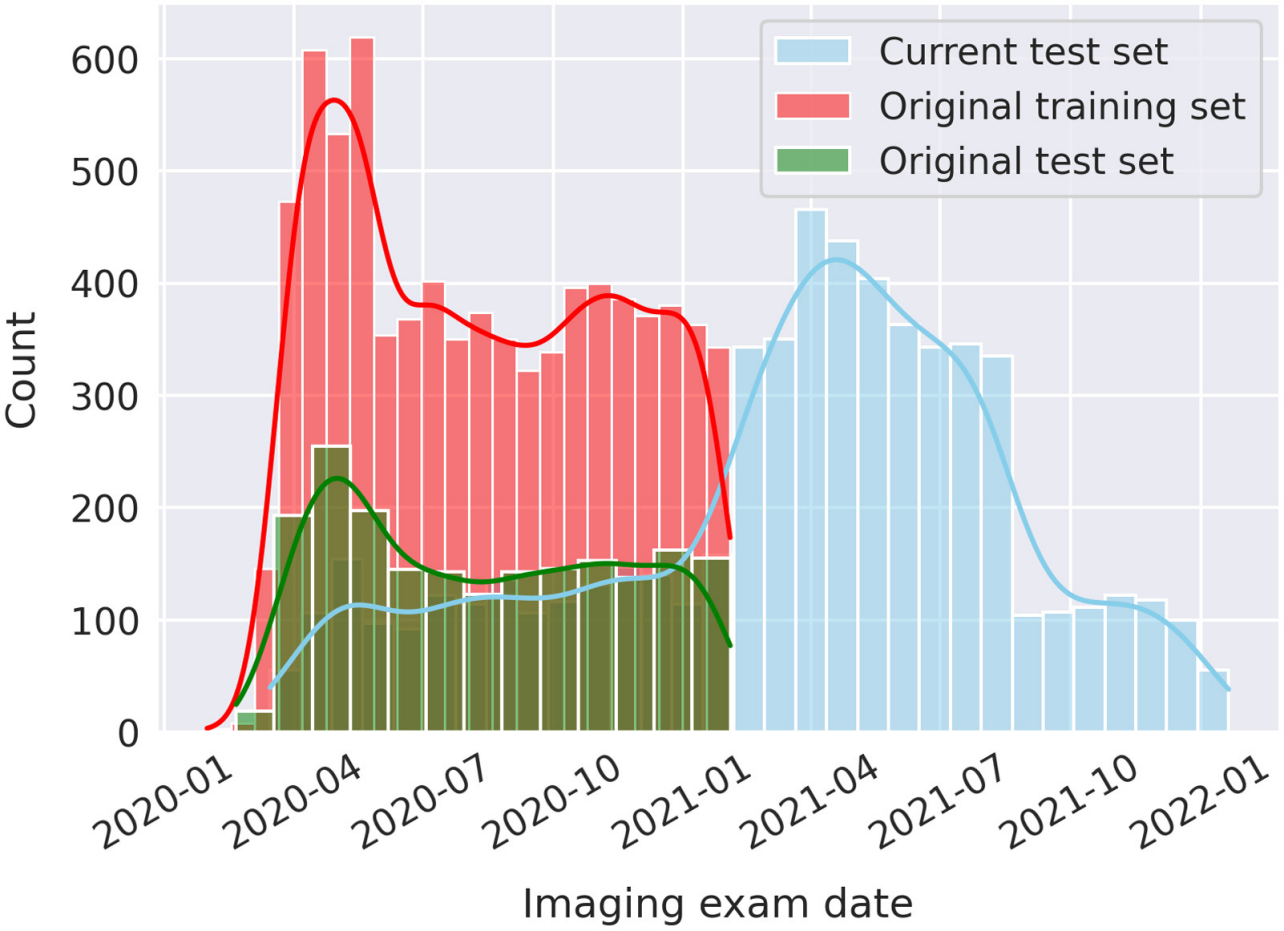
Histogram of the imaging exam dates, categorized by the current test set, the original training set, and the original test set. The current test set had a much larger date range, spanning March 15, 2020 to January 1, 2022.

The original and Alpha VOC were controlled for when the current test set was limited to the image acquisition date range of the original test set. An AUC value of 0.66 [0.62, 0.70] (significantly different from the AUC of the original test set, p<0.001) was achieved when considering cropped standard CXRs acquired within the same image acquisition dates as the original test set. This date match corresponds to the overlap of the green histogram bars with the blue as shown in Fig. 4.

#### 3.2.3 Immunization status

The current test set achieved an AUC value lower than that of the original test set when comparing the cropped standard CXRs of nonimmunized patients from the two patient cohorts, as shown in Table 6. Further, to determine whether immunization status had an impact on the DL model’s performance in classifying COVID-19 status, a comparison between immunized and nonimmunized patients in the current test set was performed; differences in AUC values failed to achieve statistical significance (p=0.60).

**Table 6:** Summary of statistical analyses performed between the original test set and the current test set. AUC comparisons were conducted using the unpaired Delong test and all three comparisons between the test sets were statistically significantly different (p<0.001).

	Original test set	Current test set
**Total comparison**		
Date range	February 20, 2020 to February 3, 2021	March 15, 2020 to January 1, 2022
Number of patients	1972	5893
COVID prevalence	15.5%	12.4%
AUC [95% CI]	0.76 [0.73, 0.79]	0.67 [0.65, 0.70]
**Date match**		
Date range	February 20, 2020 to February 3, 2021	March 15, 2020 to February 2, 2021
Number of patients (%)	1972 (100%)	1737 (29.5%)
COVID prevalence	15.5%	14.6%
AUC [95% CI]	0.76 [0.73, 0.79]	0.66 [0.62, 0.70]
**Nonimmunized patients**		
Number of patients (%)	1966 (99.7%)	4436 (75.3%)
COVID prevalence	15.5%	14.4%
AUC [95% CI]	0.76 [0.73, 0.79]	0.67 [0.65, 0.70]

#### 3.2.4 COVID severity

**Fig. 5:**
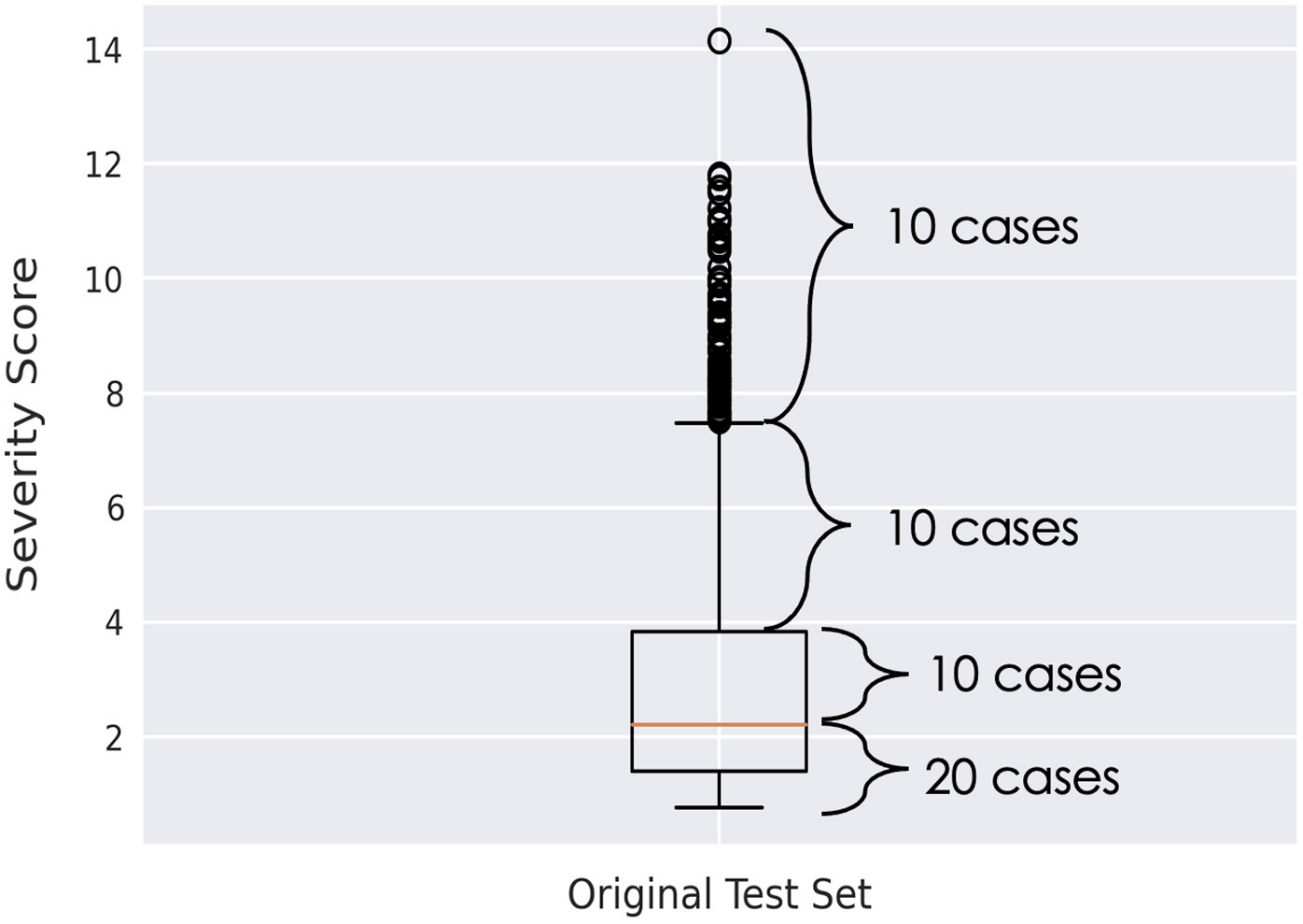
Stratified sampling of the 50 random cases from the original test set to determine the robustness of the PXS scores. 10 cases were randomly chosen from each part of the box and whisker plot to ensure an equal representation of cases from all possible PXS scores assigned.

**Fig. 6:**
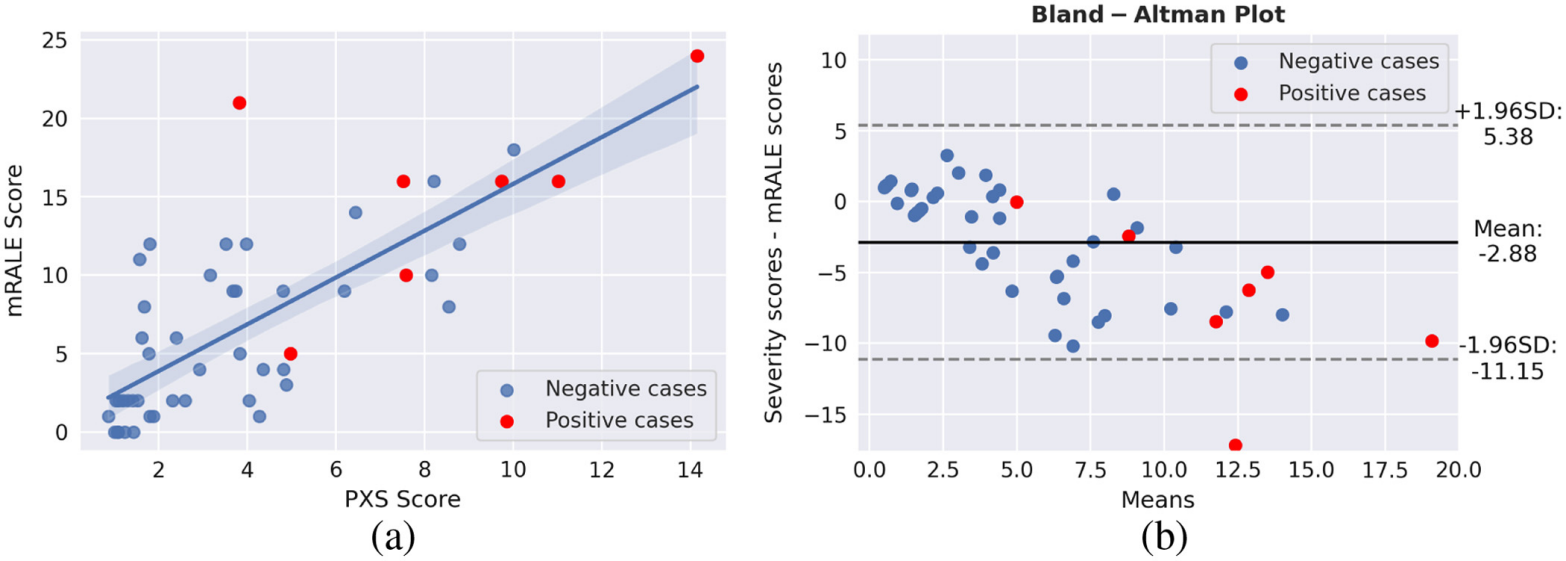
(a) A scatter plot of a subset of images (from the original test set) displaying the linear correlation between the mRALE score calculated by the radiologist and the COVID severity as determined by the DL model described by Li et al.,^24^ where the shaded blue region is the 95% CI. (b) Bland–Altman plot displaying the agreement between the methods of assessing COVID severity. The outlier outside the 95% boundary was a collapsed left lung with possible effusion.^12^

COVID severity was assessed for both patient cohorts; differences between the PXS scores failed to achieve statistical significance (p=0.17). PXS scores also failed to achieve a statistically significant difference when matching the image acquisition dates between the test sets (p=0.06). The robustness of the severity score itself was evaluated based on the 50 cases selected in Fig. 5 using the Spearman’s rank correlation coefficient (ρ=0.74, p<0.001) as well as a Bland–Altman plot to display agreement (Fig. 6). The Bland-Altman plot showed that the PXS score (calculated using the standard CXRs) was on average lower than the radiologist’s assessment [Fig. 6(b)].

**Fig. 7:**
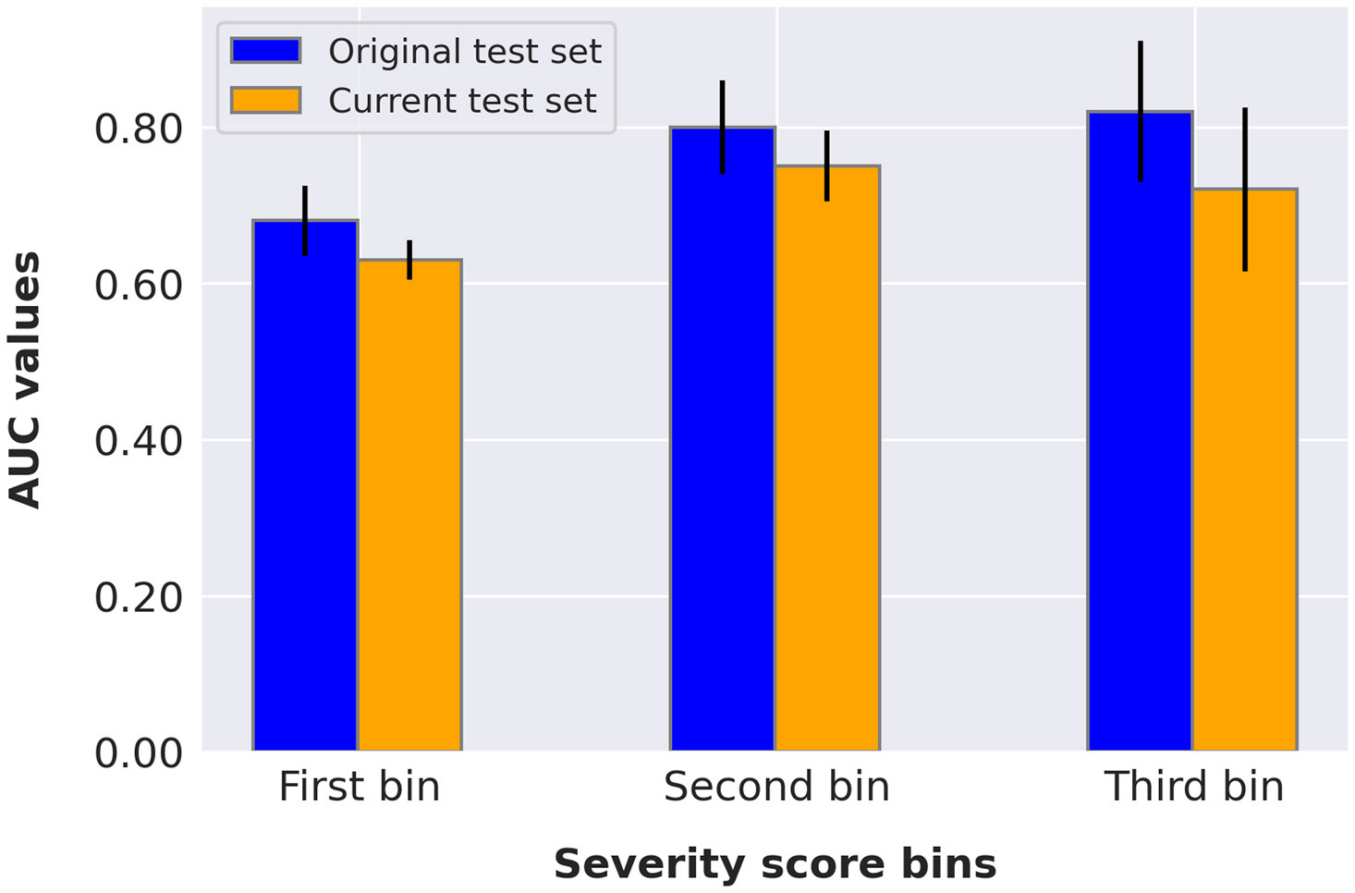
Bar plot depicting the resulting AUC values when controlling for PXS scores using histogram bins. The 95% CIs were calculated by bootstrapping the AUC values 2000 times.

**Fig. 8:**
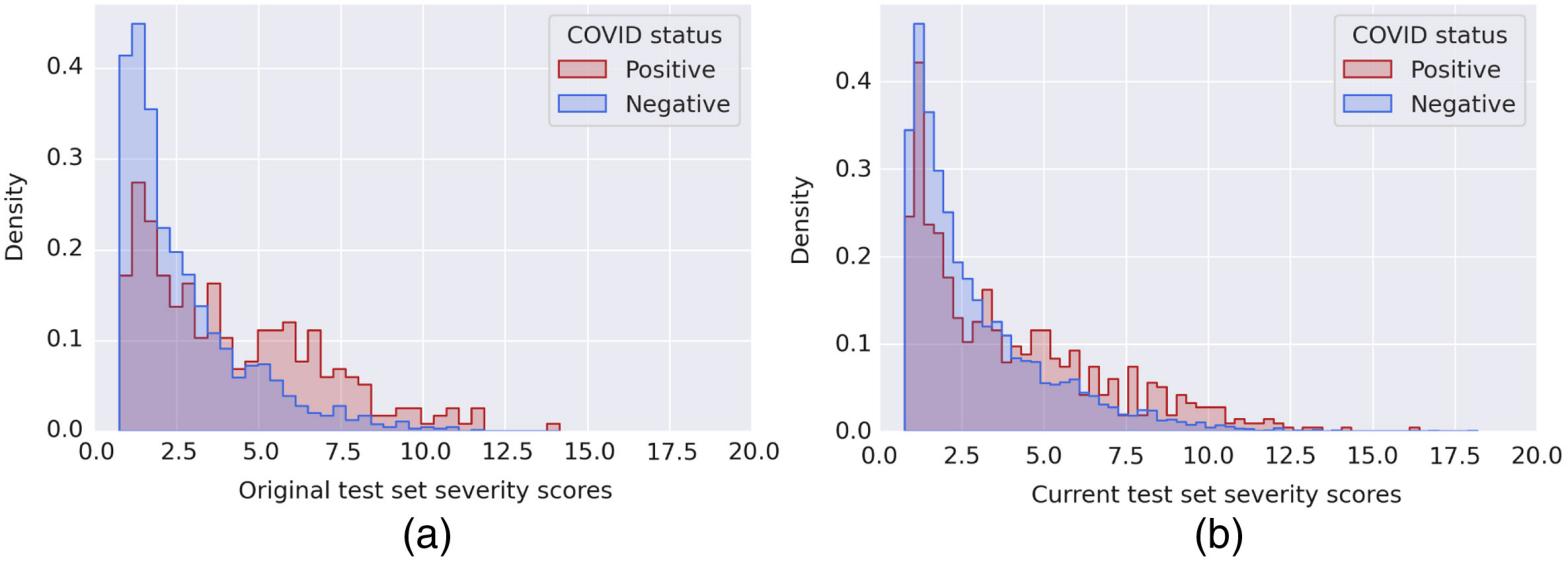
Histogram plots of the original (a) and current (b) test sets. Both distributions have strong right skews with higher frequency of positive cases having larger PXS scores.

AUC values resulting from cases in the three PXS score bins for the test sets (details presented in Table 7) are displayed in Fig. 7. The highest AUC value for the original test set was achieved for the third bin, which contained cases with the highest COVID prevalence (45.05%). The highest AUC value for the current test set resulted from the second bin, which contained cases with the second highest COVID prevalence (35.09%). Though, the 95% CIs increased with bin edges since the number of cases decreased. The COVID prevalence increased with increasing bin edges, predictably, as higher PXS scores indicate greater radiographic evidence of abnormality. Overall, the original test set yielded AUC values consistently larger than those of the current test set. The distribution of severity scores split according to positive and negative cases, per test set, is shown in Fig. 8.

**Table 7:** Definition of PXS score bins for the test sets.

	Original test set	Current test set
PXS score bin edges	0.77/4.12/7.46	0.76/5.12/9.48
Counts (*N*)	1538/343/91	4914/859/120
COVID prevalence (%)	11.31%/26.24%/45.05%	10.56%/19.79%/35.09%

#### 3.2.5 DL model predictions

**Fig. 9:**
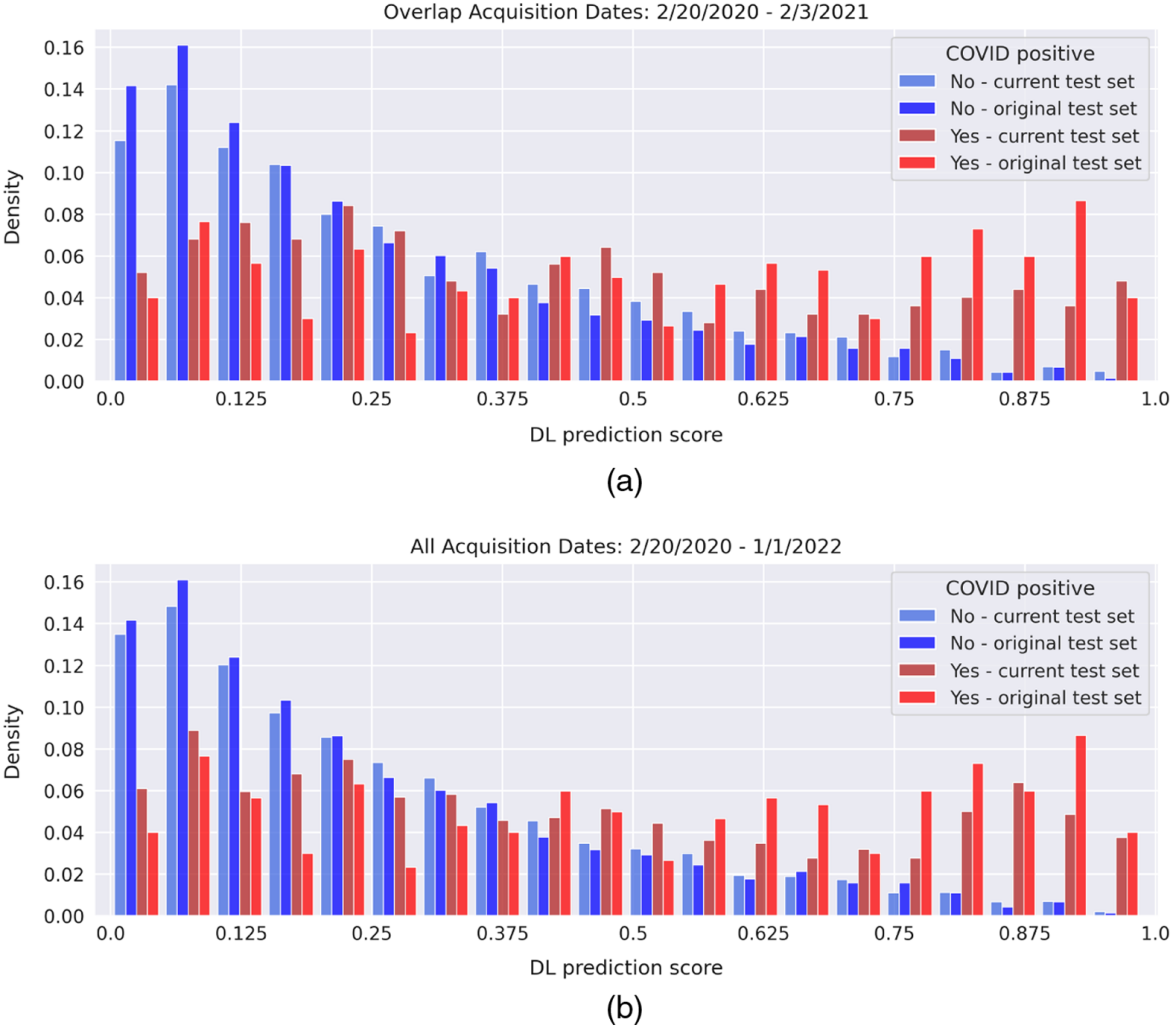
Histogram of the prediction scores of the DL model for both test sets. The distribution (a) before February 3, 2021 and (b) the entire data range. February 3, 2021 was chosen as the cutoff date as that is the last date which had an overlap of CXR acquisitions between the two patient cohorts (see Fig. 4). The histograms were normalized to have equal area.

Figure 9 presents histograms displaying the prediction scores assigned by the model for the original and current test sets. The distributions display a higher proportion of images from the original test set at low (≤0.25) and high (≥0.75) prediction scores relative to the images from the current test set, which resulted in the observed higher performance on the original test set. There was a slightly higher count for the current test set at scores in the middle of the plot, i.e., less certain predictions assigned by the DL model.

#### 3.2.6 UMAP visualization

**Fig. 10:**
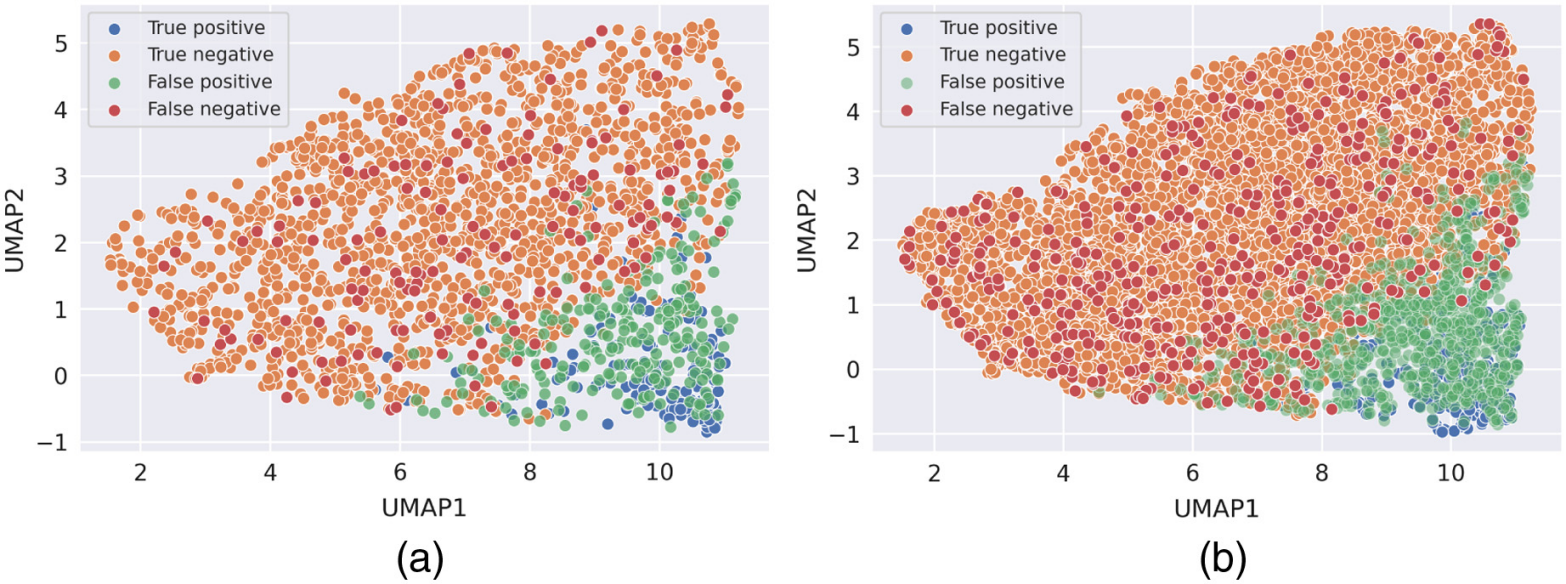
UMAP visualization of the confusion matrix for (a) the original test set and (b) the current test set. A similar decision variable was chosen by the deep net for both patient cohorts, classifying positive cases from negative cases (division between blue and orange dots). Overall, the model returned a higher percentage of TPs and lower percentage of FPs for the original test set than for the current test set.^12^

UMAP visualizations indicated that the model perceived the two sets of CXRs nearly identically (Fig. 10). This observation was supported by the MANOVA analysis, which generated an F-statistic of 1.9014 and a p-value of 0.1494, failing to achieve a statistically significant difference between the two bivariate means of the UMAPs generated for the original test set and for the current test set. However, variation existed as the percentages of true positives (TPs) and false positives (FPs) were different between the original test set (TP = 8%, FP = 11.9%) and the current test set (TP = 4.8%, FP = 12.9%).

#### 3.2.7 Patient demographics

**Fig. 11:**
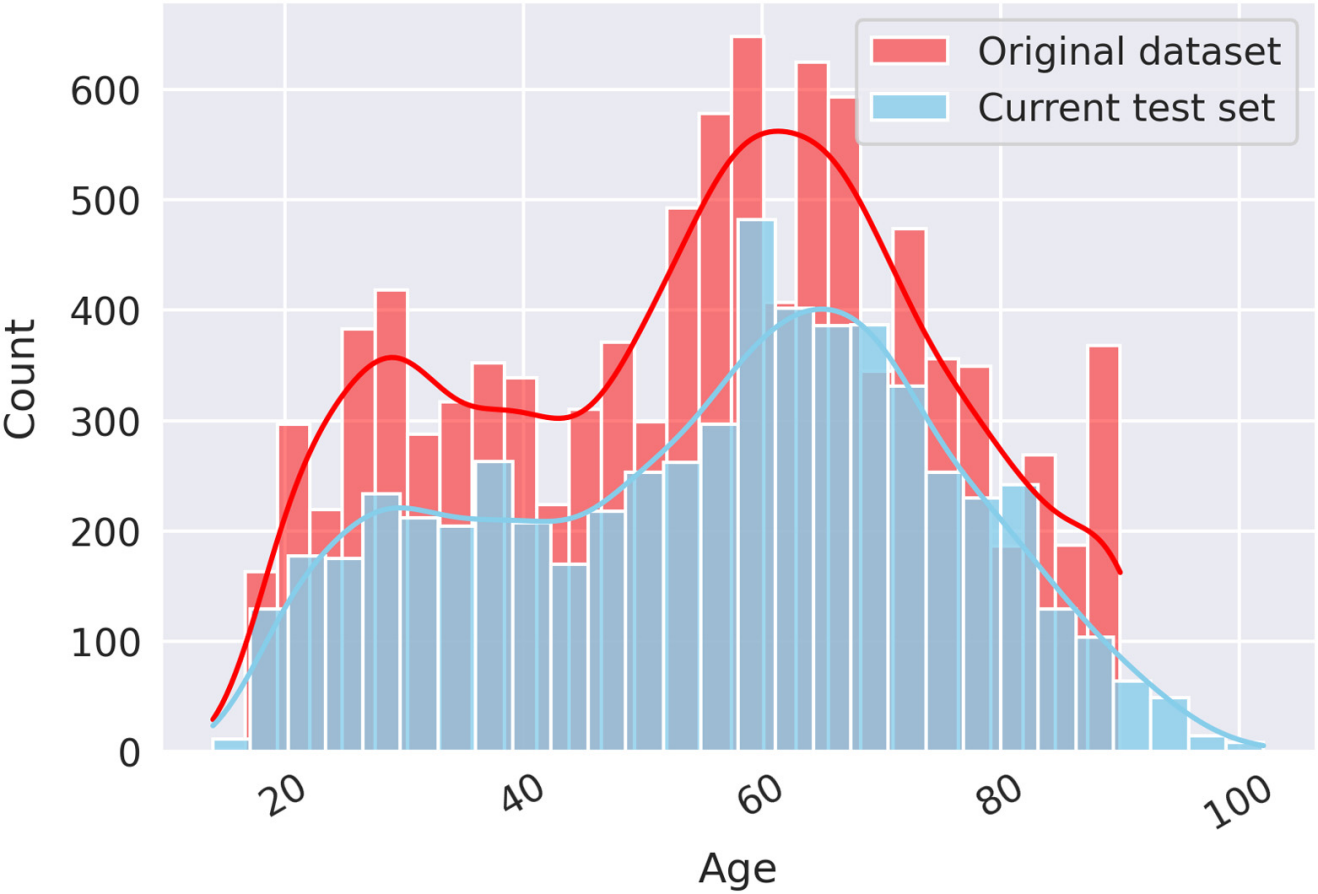
Histogram of patient age, categorized by the original dataset (which includes training, validation, and test cases) and current test set.^12^

Despite the nearly identical mean and median ages between the two test sets (Table 8), the distributions of age (Fig. 11) yielded statistically significant differences based on the Wilcoxon rank-sum test (p=0.006).

**Table 8:** Statistics of the age distributions for the original dataset (which includes training, validation, and test cases) and the current test set.

	Original dataset	Current test set
Mean age (± SD)	54.7 ± 18.9	55.9 ± 19.1
Median age (IQR)	56 (29)	59 (29)

There were more male patients than female in the original dataset [5088 men and 4772 women (52% male)] and current test set [2979 men and 2914 women (51%)]. Table 9 summarizes AUC values obtained when dividing the test sets with respect to sex. Statistical differences occurred (p<0.05) when comparing the AUC values of the original test set to the current test. There were no significant differences within each test set.

**Table 9:** Distributions of sex for the original and the current test set with their corresponding AUC values and COVID prevalence.

	AUC value	COVID prevalence
(A) Original test set — male (N=1051)	0.76 [0.71, 0.80]	14.0%
(B) Original test set — female (N=921)	0.76 [0.71, 0.80]	17.2%
(C) Current test set — male (N=2979)	0.65 [0.62, 0.69]	11.1%
(D) Current test set — female (N=2914)	0.69 [0.67, 0.72]	13.7%

#### 3.2.8 Image dimensions

When using CXRs from the original test set that were resized by the U-Net-based model to the large lung region dimensions (bottom panel in Fig. 1), the performance of the DL model decreased for all three classification algorithms relative to the published original test set results [Fig. 3(a)], obtaining AUC values of 0.74 [0.71, 0.77] for standard CXRs alone, 0.72 [0.69, 0.75] for soft-tissue CXRs alone, and 0.69 [0.66, 0.73] for both types of images. The feature fusion algorithm experienced the largest decrease in AUC values.

To determine the impact of image resizing further, the large lung region images were resized to the small lung region dimensions using the Image module from the PIL Python package. This additional resizing resulted in AUC values closer to those obtained from the original test set analysis: 0.76 [0.73, 0.79] for the standard CXRs alone, 0.72 [0.69, 0.76] for the soft-tissue CXRs alone, and 0.75 [0.72, 0.79] for both types of images. Once more, the classification algorithm incorporating both types of images experienced the largest decrease in classification performance. While the images in this additional analysis were resized to the smaller dimensions using the same Python packages as the U-Net-based model, the cropped images were not identical to the original test set as determined by evaluating the pixel values. The value of U-Net cropping was demonstrated using uncropped standard CXRs of the current test set, for which the model achieved an AUC value of 0.58 [0.56, 0.61], substantially lower than the 0.67 reported throughout this work.

## 4 Discussion

The novelty of this study involves its in-depth and exhaustive analysis of various factors that may have contributed to the significant difference in performance by the DL model between the original and current test sets. The results were explored in a variety of ways: performance was assessed across classification algorithm, by CXR exam type, and during different time periods to account for different variants of the COVID virus. Model performance was also evaluated when controlling for equipment manufacturers and various VOC. Vaccination status and disease severity were also considered to determine their impact on the classification task. In addition, patient ages and sex were taken into account, along with the model’s perception of the radiographs for the two test sets. Influence of the various cropped lung region dimensions was evaluated.

Similar to results obtained from the original test set, the cropped standard CXRs in the current test set performed better when compared with the cropped soft-tissue images. Differences in AUC values between standard and both types of CXRs, however, failed to achieve statistical significance (Table 3); however, AUC values for the three classification algorithms were all significantly lower than those of the original test set. Dividing the CXRs between portable and DES was performed to investigate whether the type of radiography unit would have an impact on robustness of the DL model. Unlike for the original test set (Table 4 in Ref. 13), there were no significant differences in AUC values as presented in Table 4. This was unexpected as the two types of CXR units generate images of different quality. For example, for soft-tissue image acquisition, a DES unit acquires an image by generating two separate energies of x-rays, and the resultant image is obtained by subtracting the two images from each other to acquire the soft-tissue image. Portable units generate x-rays only at one energy followed by postprocessing algorithms, which create a synthetic soft-tissue image. Further, patient geometry is different between the two types of units, as patients are typically oriented in anterior-posterior positioning for portable units and posterior-anterior for DES units. One must also be aware of the motion artifacts that arise from a CXR acquired from a portable unit. Despite these factors, no significant differences were observed in the AUC values between the two types of radiography units.

Given that there were no significant differences in AUC values between the two types in this work, however, this factor did not explain the decrease in model performance. The original and current test sets were also visually assessed, and there were no apparent differences in image quality between the two sets. Further, gradient-weighted class activation mapping (Grad-CAM) heatmaps were employed to visually assess the predictions of the model for both negative and positive cases from the original and current test sets for high (>10) and low (<1) severity as determined by the PXS score. This analysis, however, did not portray a distinction between the two test sets.

Analysis of patient demographics for the two sets, i.e., age and sex distributions, did not provide any further explanations regarding the discrepancy of performance. The findings when matching for sex were consistent with the other investigations: the model better classified COVID-19 status of patients from the original test set than from the current.

The original test set had more “obvious” negative and positive cases than the current test set (Fig. 9), which may have impacted the differences in performance between the two test sets. The “obviousness” of a case was suggested by the increased counts for the low (cases the model perceived as negative) and high (cases the model perceived as positive) prediction scores for the original test set when compared with the current test set [Fig. 9(b)]. This trend was amplified when limiting the date range prior to February 3, 2021 [Fig. 9(a)], i.e., limiting the current test set to the image acquisition date range of the original test set (Sec. 3.2.2), which likely explains why the date range-matching analysis did not increase the AUC value to one comparable to that of the original test set. Immunization status also did not provide an explanation for the discrepancy in decreased performance.

While the PXS score was designed to evaluate only COVID-positive patients, there was merit in applying this technique to the CXRs of COVID-negative patients because patients obtaining a CXR usually have suspicion of some abnormality in the lung. Therefore, the PXS score, which is defined as the median Euclidean distance between the image of interest and “normal” images, was still a useful metric to incorporate because many of the CXRs of the COVID-negative patients were not “normal.”

The data-reduction capability offered by the UMAP was applied to the penultimate global average pooling layer; a two-dimensional embedding was generated that helped visualize how the DL model interpreted the CXRs, thus providing an interpretation of the perception of the radiographs by the model. A nearly identical embedding for images acquired from both cohorts was illustrated. COVID-negative patients appeared to be more sparse in the embedding for the current test set, however, than for the original test set.

While this work followed the same image preprocessing for the original dataset and current test set, investigation in changes of the cropped lung region dimensions provided insight on how small changes may lead to different results when using DL models. Overall, this work demonstrated the complexity of attaining model robustness and generalizability: an “off-the-shelf” deep net capable of performing classification tasks across different datasets with minimal training remains an elusive task. Therefore, one explanation for the significant difference in performance between the test sets used in this study is a lack of generalizability of the model, which was unable to correctly classify COVID for a new test set (from the same institution) as robustly as it did for the data on which it had been trained originally.

To address this lack of generalizability, future work will investigate (1) the impact of patient demographics and clinical factors on the classifier, (2) whether there were differences in the “obvious” negative or positive patients between the two cohorts, and (3) altering the architecture of the model to make it more robust. First, while patient age and sex was examined for both the original dataset and current test set, matching for age and sex on the training set and test set could provide an explanation for the decrease in performance of the model (e.g., investigate the higher AUC value obtained when considering female patients on the current test set further). COVID severity could also be controlled for between the training and test set. Second, an analysis of the characteristics of only the positive patients from both cohorts will be performed. For example, if the positive patients in the original test set are older than the positive patients in the current test set, then disease presentation across age may contribute to the performance decrease. Third, a weight regularizer can be applied to the DenseNet layers (specifically, L2 regularization) to impose a penalty on the calculated weights, which in turn will prevent model overfitting and possibly make it more generalizable. This will be one approach in potential ablation studies. Repartitioning the original dataset will also be done to determine whether favorable partitions were the reason for discrepancy in performance. Overall, the regularization could also mitigate the impacts of random data partitions, yielding more robust results.

While the the AI community joined in on the efforts early in the pandemic, the community also started recognizing the shortcomings of the methodologies employed, leading to unreliable models.^9^^,^^33^ For example, training sets early in the pandemic suffered from small sizes and class imbalances, which made it unlikely that the results of AI models would generalize to broader populations. Goncalves et al.^34^ reported that some models originally trained on small datasets from China were intended for use in European populations, resulting in ineffective models due to differences in patient biomarkers and data acquisition protocols. Further, many public datasets of COVID-19 patients comprised images taken from journal articles without access to the original DICOM images,^35^ raising concerns about image quality and whether “pictures of pictures” provide the same quality data as original images.^33^^,^^36^^,^^37^ What distinguishes this research, however, is the systematic analyses performed to compare datasets that were acquired from the same institution, using the same machinery and imaging protocols; therefore, this novel work provides invaluable insight to how DL models may falter even within the same institution, which in turn can reveal ways to mitigate the lack of model robustness.

## 5 Conclusion

A larger and more current test set of CXRs was used to validate the performance of a pre-trained DL model designed to differentiate COVID-positive from COVID-negative patients. An AUC value of 0.67 for cropped standard CXRs, 0.65 for cropped soft-tissue CXRs, and 0.67 for both types of cropped images were achieved, which were significantly lower than initial performances of 0.76, 0.73, and 0.78, respectively, on the original test set. Several factors were considered to determine their impact on the observed differences in model performance on the test sets, including time period of image acquisition, immunization status, age and sex distribution, and disease severity. The underperformance of the model on the current test set may be explained by a lack of model generalizability.

## Data Availability

A collection of data presented in this article are now publicly available at https://data.midrc.org/. The archived version of the code described in this manuscript can be freely accessed through GitHub at https://github.com/MIDRC/CRP10/blob/main/COVID-19_CXR_binary_classification.py.
